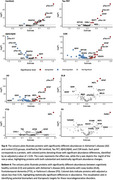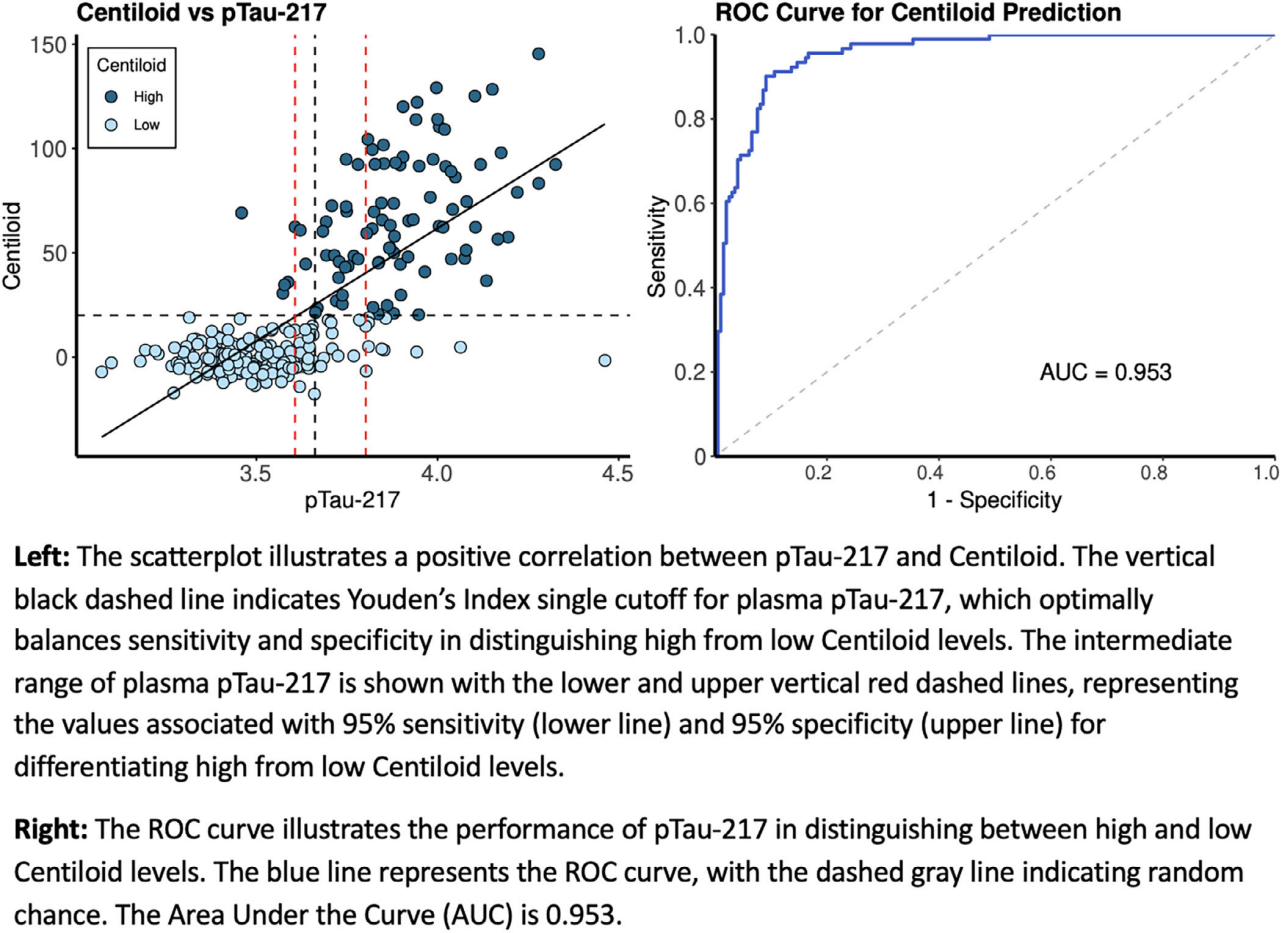# Decoding Neurodegenerative Disease with NULISA: Disease‐Specific Biomarkers and Predictive Signatures Unveiled

**DOI:** 10.1002/alz70856_101191

**Published:** 2025-12-24

**Authors:** Katherine Gong, Jigyasha Timsina, Muhammad Ali, Yike Chen, Menghan Liu, Ciyang Wang, Maulikkumar P Patel, Gyujin Heo, Tammie L.S. Benzinger, Suzanne E. Schindler, John C. Morris, David M. Holtzman, Laura Ibanez, Carlos Cruchaga

**Affiliations:** ^1^ Department of Psychiatry, Washington University School of Medicine, St. Louis, MO, USA; ^2^ NeuroGenomics and Informatics Center, Washington University School of Medicine, St. Louis, MO, USA; ^3^ Division of Biology and Biomedical Sciences, Washington University in St. Louis, St. Louis, MO, USA; ^4^ Hope Center for Neurological Disorders, Washington University School of Medicine, St. Louis, MO, USA; ^5^ Knight Alzheimer Disease Research Center, Washington University School of Medicine, St. Louis, MO, USA; ^6^ Department of Radiology, Washington University School of Medicine, St. Louis, MO, USA; ^7^ Department of Neurology, Washington University School of Medicine, St. Louis, MO, USA; ^8^ Hope Center for Neurological Disorders, Washington University in St. Louis, St. Louis, MO, USA; ^9^ Knight Alzheimer Disease Research Center, St. Louis, MO, USA; ^10^ Department of Genetics, Washington University School of Medicine, St Louis, MO, USA; ^11^ The Charles F. and Joanne Knight Alzheimer Disease Research Center, St Louis, MO, USA; ^12^ Washington University School of Medicine, St. Louis, MO, USA

## Abstract

**Background:**

Highly sensitive plasma assays enable accurate blood‐based neurodegeneration biomarkers, offering minimally invasive options for routine clinical use. This study quantified 123 plasma proteins using the Nucleic acid Linked Immuno‐Sandwich Assay (NULISA) platform in over 3,000 individuals with AD, DLB, FTD, PD and controls. We aimed to identify general neurodegenerative as well as disease‐specific biomarkers.

**Method:**

We included 3,668 plasma samples from 3,002 Knight ADRC participants [1,092 AD, 39 FTD, 28 DLB, 9 PD, and 1,579 controls]. Differential abundance, ROC, and AUC analyses were performed in each disease separately, as well as using amyloid PET, tau PET, CSF Aβ42/Aβ40 ratios, and CDR as quantitative traits. GMM clustering was applied to determine protein cutoffs. Survival analyses were performed to identify proteins associated with progression to symptomatic AD. Gene Ontology enrichment analysis was conducted to explore the biological significance of the identified proteins. All analyses were corrected by multiple test correction using FDR.

**Result:**

Among the 123 protein analytes measured, 78 were significantly associated with AD, two with DLB, two with FTD, and one with PD. Eight analytes were associated with amyloid Centiloid PiB levels, seven with tau PET imaging, 14 with CSF Aβ42/Aβ40 ratios, and 73 with CDR. Plasma pTau217, adjusted for age and sex, achieved an AUC of 0.81 (95% CI: 0.79‐0.83) for clinical AD and 0.95 (95% CI: 0.93‐0.98) for amyloid positivity. Using the double‐standard cutoff approach, we achieved a plasma pTau217 and Centiloid concordance rate of 93.59%. Pathway analysis revealed distinct pathways enriched in specific diseases. For example, receptor and ligand binding/signaling pathways, including vascular endothelial growth factor receptor binding (*p* = 3.21 × 10^−4^), were uniquely associated with AD, primarily influenced by VEGFD and VEGFA.

**Conclusion:**

Our study is a comprehensive analysis of central nervous system and inflammatory proteins measured in plasma using NULISA in the context of AD. It validates the predictive accuracy of plasma pTau217 and its strong correlation with Centiloid levels and other neuroimaging biomarkers when measured with this novel platform. These findings further establish the potential of NULISA quantification as a reliable tool for research and clinical applications in neurodegenerative diseases.